# Testing rare variants for hypertension using family-based tests with different weighting schemes

**DOI:** 10.1186/s12919-016-0036-7

**Published:** 2016-10-18

**Authors:** Xuexia Wang, Xingwang Zhao, Jin Zhou

**Affiliations:** 1 Department of Mathematics, University of North Texas, Denton, TX 76203 USA; 2 Joseph J. Zilber School of Public Health, University of Wisconsin–Milwaukee, Milwaukee, WI 53205 USA; 3 Division of Epidemiology and Biostatistics of Mel and Enid Zuckerman College of Public Health, University of Arizona, 1295 N. Martin Ave., Campus PO Box: 245211, Drachman Hall A242, Tucson, AZ 85724 USA

## Abstract

Next-generation sequencing technology makes directly testing rare variants possible. However, existing statistical methods to detect common variants may not be optimal for testing rare variants because of allelic heterogeneity as well as the extreme rarity of individual variants. Recently, several statistical methods to detect associations of rare variants were developed, including population-based and family-based methods. Compared with population-based methods, family-based methods have more power and can prevent bias induced by population substructure. Both population-based and family-based methods for rare variant association studies are essentially testing the effect of a weighted combination of variants or its function. How to model the weights is critical for the testing power because the number of observations for any given rare variant is small and the multiple-test correction is more stringent for rare variants. We propose 4 weighting schemes for the family-based rare variants test (FBAT-v) to test for the effects of both rare and common variants across the genome. Applying FBAT-v with the proposed weighting schemes on the Genetic Analysis Workshop 19 family data indicates that the power of FBAT-v can be comparatively enhanced in most circumstances.

## Background

Hypertension or high blood pressure is a chronic medical condition with unknown complex etiology [1]. Blood pressure is summarized by 2 measurements: systolic and diastolic. High blood pressure is said to be present if blood pressure (BP) is 140 mm Hg systolic or higher (SBP) or 90 mm Hg diastolic or higher (DBP). More than 1 billion people worldwide have hypertension [2], which is a major risk factor for stroke, myocardial infarction, heart failure, and is a cause of chronic kidney disease [3–5]. Both genetic and environmental factors are likely to contribute to this disease. Ehret et al. conducted a large-scale genome-wide association study of hypertension in 2011 and identified 10 novel loci related to BP physiology [6]. Although numerous common genetic variants with small effects on BP have been identified [6–8], the identified variants account for only a small fraction of disease heritability [9]. One potential source of missing heritability is the contribution of rare variants. Next-generation sequencing technology allows sequencing the whole genome of large groups of individuals, thus making direct testing of rare variants feasible.

However, existing statistical methods to detect common variants may not be optimal for detecting rare variants because of allelic heterogeneity and the extreme rarity of individual variants [10]. Recently, several statistical methods to detect associations of rare variants were developed, including both population-based and family-based methods. Compared with population-based methods, family-based methods have more power and can prevent bias induced by population substructure [11]. However, family-based methods to detect rare variants are not well established, which may be the result of the difficulties and complexities in testing rare variants in pedigree data. Let *x*_*im*_ denote the genotype (number of minor alleles) of the *i* ^*th*^ individual at the *m* ^*th*^ variant. Both population-based and family-based methods for rare variant association studies are essentially testing the effect of a weighted combination of variants, ∑_*m*_ *w*_*m*_ *x*_*im*_, or its function. How to model the weights *w*_*m*_ is critical for the testing power because the number of observations for any given rare variant is small and the multiple-test correction is more stringent for rare variants [12]. Family-based association tests for rare variants (FBAT-v) is a recently developed family-based method. In their paper on FBAT-v, De et al. used a weighting scheme based on allele frequency and noted that the optimal weighting scheme is unknown and dependent on the underlying disease model [11]. To powerfully test rare variants using family-based tests for hypertension based on the Genetic Analysis Workshop 19 (GAW19) data and to provide a powerful means to test the rare variants that play an important role in a disease etiology, we propose and evaluate 4 weighting schemes for the FBAT-v.

The GAW19 data consists of a whole genome sequencing data set for a large-scale pedigree-based sample that includes 959 individuals. Among the 959 individuals, 849 have simulated phenotypes in all 200 replicates. Our analysis focuses on the family data of the 849 individuals. We applied FBAT-v with the proposed weighting schemes to the GAW19 family data. Our results indicate that the type I error rates for all the methods compared are under control and the power of the FBAT-v with an optimal weight outperforms the methods compared in most circumstances.

## Methods

Consider a sample of *n* trios. Each individual in the sample has been genotyped at *M* variants in a genomic region. Denote *y*_*i*_ as the quantitative trait for the *i* ^*th*^ offspring. Denote *X*_*i*_ = (*x*_*i*1_, …, *x*_*iM*_)^*T*^ as the genotypic score of the *i* ^*th*^ individual, where *x*_*im*_ ∈ {0, 1, 2} is the number of minor alleles that the *i* ^*th*^ individual has at the *m* ^*th*^ variant.

Suppose that we have *p* covariates. Let (*z*_*i*1_, …, *z*_*ip*_)^*T*^ denote covariates of the *i* ^*th*^ individual. We adjust both trait value *y*_*i*_ and genotypic score *x*_*im*_ for the covariates by applying linear regressions. That is,

$$ {y}_i={\alpha}_0+{\alpha}_1{z}_{i1}+\dots +{\alpha}_p{z}_{ip}+{\varepsilon}_i\kern1em \mathrm{and}\kern1em {x}_{im}={\alpha}_{0m}+{\alpha}_{1m}{z}_{i1}+\dots +{\alpha}_{pm}{z}_{ip}+{\tau}_{im} $$

Let *ỹ*_*i*_ and ${\tilde{x}}_{im}$ denote the residuals of *y*_*i*_ and *x*_*im*_, respectively. Denote ${\tilde{X}}_i=\left({\tilde{x}}_{i1},\dots, {\tilde{x}}_{iM}\right)$ as the residuals of the genotypic score of the *i* ^*th*^ individual.

To test the null hypothesis of no association, the weighted family-based association test (FBAT) statistic for rare variants (FBAT-v) [11] can be defined as

1 $$ Z={W}^{(w)}/\sqrt{Var\left({W}^{(w)}\right)} $$

$$ \begin{array}{l}\mathrm{where}\ {W}^{(w)}={\displaystyle \sum_{m=1}^M{w}_m{U}_m},\ {U}_m={\displaystyle \sum_{i=1}^n\left({\tilde{y}}_i-\mu \right)\Big({\tilde{x}}_{im}-E\left({\tilde{x}}_{im}\Big|{P}_{im}\right)},\ \mu\ \mathrm{is}\ \mathrm{the}\ \mathrm{mean}\ \mathrm{o}\mathrm{f}\ \mathrm{the}\ \mathrm{trait}\ \mathrm{and}\ {P}_{im}\kern0.5em \mathrm{is}\ \mathrm{the}\ \mathrm{parental}\\ {}\mathrm{genotypes}\ \mathrm{f}\mathrm{o}\mathrm{r}\ \mathrm{the}\ {i}^{th}\ \mathrm{f}\mathrm{amily}\ \mathrm{o}\mathrm{f}\ \mathrm{the}\ {m}^{th}\ \mathrm{variant}.\end{array} $$

*Z* approximately follows *N*(0,1) in large samples under the null hypothesis of no association. For large pedigrees, pedigrees are decomposed into nuclear families which are treated as independent unless a trait locus is known to be linked to the markers under test. The pedigree’s contribution to the tests statistic is obtained by summing over all nuclear families within the pedigree. However, in the case where linkage is present and the null hypothesis states “no association but linkage presents,” the genotypes of the different nuclear families derived from 1 pedigree are correlated. In this case, the variance of *W* ^(*w*)^ should be computed empirically in order to keep the type I error rates under control.

To increase the power of FBAT-v, we propose the following 3 weighting schemes that use only founders’ information. In the family data, founders are individuals who have no parents specified (assuming *l* founders in total): genotype risk value *(grv)*; log odds ratio *(lor)*; and optimal weight *(ow)*.

### Genotype risk value

The *genotype risk value (grv)* weighting scheme is based on genotype frequencies and effect sizes for each single-nucleotide polymorphism (SNP) from the single-variant study. Comparing the continuous trait with a prespecified threshold, we can classify subjects into 2 categories: cases and controls. Assume that AA, AB, and BB (A, low-risk allele; B, high-risk allele) are genotypes for a SNP. Based on the log-additive model, the 3 genotypes have a relative risk of 1, odds ratio (OR), OR^2^, respectively. If the B allele has frequency *p*, then the average relative risk (ARR) in the population can be calculated as: ARR = (1 − *p*)^2^ + 2*p*(1 − *p*)OR + *p* ^2^OR^2^. 1/ARR, OR/ARR, and OR^2^/ARR are assigned as weights for genotype AA, AB, and BB, respectively. This weighting scheme puts big weights on variants with more copies of the risk alleles.

### Log odds ratio

The *log odds ratio (lor)* weighting scheme is based on effect size for each SNP from the single-variant study. Similarly, comparing the continuous trait with a prespecified threshold (eg, SBP = 140 mm Hg), we can classify subjects into 2 categories: cases and controls. We can then build a logistic regression model by adjusting for covariates such as age, gender, and smoking status. The weight for each individual SNP is determined by the lor of its association with disease. This weighting scheme puts big weights on variants with large effect sizes associated with disease.

### Optimal weight

The *optimal weight (ow) w*_*s*_^*o*^ was first proposed by Sha et al. [13] in a population-based test TOW (test for testing the effect of an optimally weighted combination of variants) by assuming the independence among rare variants:

${w}_m^o=\rho \left(\tilde{y},{\tilde{x}}_m\right)/\sqrt{{\displaystyle \sum_{i=1}^l{\left({\tilde{x}}_{im}-{\overline{\tilde{x}}}_m\right)}^2}},$ where *ρ* is the correlation coefficient between trait value residuals *ỹ* of unrelated individuals and genotypic score residuals. This weighting scheme gives large weights to rare variants that have small allele frequencies and strong associations with the trait of interest.

All the aforementioned weighting schemes—*grv*, *lor*, and *ow—*can adjust the direction of the association, which potentially can boost power of FBAT-v as FBAT-v only focuses on all the variants having effects in the same directions and can lead to lower power when there are variants with effects in the opposite directions. In addition to the aforementioned weighting schemes, we also propose to use functional prediction scores as weights in FBAT-v. By incorporating computational predictions of the functional effects of nonsynonymous variants into FBAT-v, it can avoid the loss of power that results from combining both functional and nonfunctional variants [12].

### Functional prediction

Price et al. [12] first incorporated computational predictions of the functional effects of missense variants in their statistical test and reported that incorporating computational predictions of functional importance further boosted power. In this study, we investigate whether incorporation of PolyPhen-2 scores as weights improves the FBAT-v test. We use SnpEff (http://snpEff.sourceforge.net) [14] to predict nonsynonymous, splice, and stop variants, and to obtain the predicted functional scores of all nonsynonymous SNPs with PolyPhen-2 algorithm (http://genetics.bwh.harvard.edu/pph2) [15]. We assign weights equal to the PolyPhen-2 score for nonsynonymous SNPs and 0 otherwise. For those nonsynonymous SNPs without a prediction score, we impute them with the corresponding median prediction score of a gene.

## Results

We apply the FBAT-v method incorporating the proposed weighting schemes—*grv, lor, ow,* and functional prediction *(fp)*—to the GAW19 family data, which consists of a whole genome sequencing data set for a large-scale pedigree-based sample of 959 individuals. Among the 959 individuals, 849 have simulated phenotypes in all 200 replicates. Our analysis focuses on the family data of the 849 individuals. The genotype, phenotype, and other information of 108 founders (unrelated individuals) out of the 849 individuals are used to calculate the *grv, lor,* and *ow* weights.

To evaluate type I error rates and the power of FBAT-v incorporating the 4 different weighting schemes, we employ the complete set of 200 replicates in the GAW19 family data. Significance is assessed in significance levels of 0.1, 0.05, and 0.01. For power comparisons, FBAT-v with the 4 weighting schemes is compared with FBAT-v with the weighting scheme based on allele frequencies as in De et al. [11]. In our analyses, we focus on 2 continuous phenotypes: SBP and DBP. The estimated heritability for SBP and DBP is in the range of 20–30 % [16]. We apply linear regression for each exam by adjusting for age, sex, and BP meds (ie, current use of antihypertensive medications) to generate standardized residuals for traits and genotypic scores. Our final analysis is based on the average residuals over 3 examinations.

### Type I error

To estimate the type I error rates, using our proposed approaches, we test association between the simulated trait Q1 and a region including 28 SNPs selected from gene *MAP4* of chromosome 3. Q1 in the family data set is simulated as a normally distributed quantitative trait that is correlated among family members (additive genetic heritability = 0.68), but not influenced by any of the genotyped SNPs. Therefore, any observed associations are false positives. Table 1 shows the type I error rate of FBAT-v with 5 different weighting schemes: FBAT-v with the weighted sum weights as in De et al. [11] using empirical variance (FBAT-v-e); FBAT-v with *grv* weights using empirical variance (FBAT-v-e-grv); FBAT-v with *lor* weights using empirical variance (FBAT-v-e-lor); FBAT-v with *ow* weights using empirical variance (FBAT-v-e-ow); and FBAT-v with *fp* weights using empirical variance (FBAT-v-e-fp). The type I error rates of FBAT-v with all the 5 weighting schemes are under control.

**Table 1:** Type I error of FBAT-v with different weighting schemes and using trait Q1

Significance level (95 % CI)	Tests				
	FBAT-v-e	FBAT-v-e-grv	FBAT-v-e-lor	FBAT-v-e-ow	FBAT-v-e-fp
0.01 (−0.004, 0.024)	0.005	0.01	0.0	0.015	0.015
0.05 (0.02, 0.08)	0.065	0.060	0.05	0.065	0.055
0.1 (0.058, 0.142)	0.11	0.1	0.085	0.12	0.095

*CI* confidence interval, *FBAT-v-e* FBAT-v with weighted sum weights as in De et al. [11] using empirical variance, *FBAT-v-e-fp* FBAT-v with functional prediction *(fp)* weights using empirical variance, *FBAT-v-e-grv* FBAT-v with genotype risk value *(grv)* weights using empirical variance, *FBAT-v-e-lor* FBAT-v with log odds ratio *(lor)* weights using empirical variance, *FBAT-v-e-ow* FBAT-v with optimal weight *(ow)* weights using empirical variance

### Power comparison

To evaluate the performance of our approaches, we compare the power of FBAT-v-e, FBAT-v-e-grv, FBAT-v-e-lor, FBAT-v-e-ow, and FBAT-v-e-fp in detecting association between SBP or DBP and the top 5 genes (*MAP4*, *TNN*, *NRF1*, *LEPR*, and *FLT3*) influencing simulated SBP or DBP.

Genes were defined by transcription start and end positions obtained from the University of California Santa Cruz (UCSC) Genome Browser hg19 build (http://genome.ucsc.edu/). Table 2 shows the summary statistics of the top 5 genes that influence simulated SBP and DBP. The total variance explained by each of the 5 genes is greater than 1 %. The percentage of rare variants in each gene is more than 50 % with a minor allele frequency (MAF) of less than 5 %, and greater than 38 % with a MAF of less than 1 %.

**Table 2:** Summary statistics of the top 5 genes

Gene	CHR	Position	No. of SNPs	No. of FV	MAF <1 %	MAF <5 %	TVE (%)
*MAP4*	3	(47892180, 48130769)	894	15	621(69.46 %)	740(82.71 %)	6.48
*TNN*	1	(175036994, 175117202)	533	18	224(42.03 %)	274(51.41 %)	4.08
*NRF1*	7	(129251555, 129396922)	740	14	385(51.33)	489(66.08 %)	2.65
*LEPR*	1	(65886335, 66103176)	980	8	380(38.78 %)	516(52.65 %)	2.5
*FLT3*	13	(28577411, 28682904)	849	10	340(40.04 %)	488(57.48 %)	1.22

*CHR* chromosome, *FV* functional variants, *MAF* minor allele frequency, *TVE* total variance explained

Table 3 indicates power of the compared methods for association study in each gene. Because *MAP4* can explain 6.48 % of the disease heritability and it has 69.46 % of rare variants with a MAF of less than 1 %, all the compared methods have medium power in detecting *MAP4.* In general, compared with FBAT-v-e by only considering allele information, FBAT-v-e-ow performs better with additional genetic effect information obtained from the unrelated individuals. The power of all the methods is pretty small for all other 4 genes. Two of the possible reasons are the ability to explain the lower percentage of rare variants (<51 %) and the lower heritability (<5 %). We did notice that FBAT-v-e-fp performs better in gene *TNN* than other methods. One possible reason is that there are more functional variants residing on this gene.

**Table 3:** Power comparisons of FBAT-v with different weighting schemes for SBP/DBP

Gene	Power of Tests				
	FBAT-v-e	FBAT-v-e-grv	FBAT-v-e-lor	FBAT-v-e-ow	FBAT-v-e-fp
Significance level = 0.05					
*MAP4*	0.63/0.505	0.65/0.53	0.51/0.47	0.68/0.56	0.65/0.52
*TNN*	0.015/0.03	0.01/0.03	0.02/0.01	0/0.02	0.06/0.04
*NRF1*	0/0.025	0/0.01	0.01/0	0.03/0.02	0.05/0.03
*LEPR*	0.035/0.04	0.04/0.01	0.03/0.01	0.05/0.03	0.04/0.035
*FLT3*	0/0.025	0.02/0.04	0/0.01	0.04/0.05	0/0.03
Significance level = 0.1					
*MAP4*	0.82/0.715	0.86/0.74	0.71/0.62	0.85/0.765	0.83/0.74
*TNN*	0.075/0.085	0.08/0.1	0.09/0.115	0.1/0.16	0.22/0.19
*NRF1*	0.035/0.1	0.03/0.125	0.02/0.09	0.06/0.185	0.08/0.145
*LEPR*	0.07/0.105	0.05/0.09	0.08/0.135	0.11/0.2	0.1/0.12
*FLT3*	0.035/0.1	0.05/0.13	0.04/0.11	0.055/0.15	0.04/0.13
Significance level = 0.2					
*MAP4*	0.90/0.865	0.92/0.87	0.85/0.77	0.95/0.89	0.94/0.85
*TNN*	0.17/0.2	0.15/0.22	0.18/0.21	0.15/0.255	0.29/0.33
*NRF1*	0.155/0.195	0.18/0.21	0.14/0.18	0.2/0.24	0.17/0.22
*LEPR*	0.07/0.165	0.08/0.175	0.09/0.19	0.14/0.22	0.11/0.17
*FLT3*	0.135/0.195	0.15/0.18	0.17/0.22	0.195/0.17	0.15/0.215

*FBAT-v-e* FBAT-v with weighted sum weights as in De et al. [11] using empirical variance, *FBAT-v-e-fp* FBAT-v with functional prediction *(fp)* weights using empirical variance, *FBAT-v-e-grv* FBAT-v with genotype risk value *(grv)* weights using empirical variance, *FBAT-v-e-lor* FBAT-v with log odds ratio *(lor)* weights using empirical variance, *FBAT-v-e-ow* FBAT-v with optimal weight *(ow)* weights using empirical variance

## Discussion and conclusions

In this article, we propose 4 weighting schemes for the FBAT-v. Simulation studies indicate that both the *grv* and *ow* weighting schemes can boost FBAT-v’s power when the proportion of rare variants is large (eg, 69 % with a MAF of <1 %) and total variance being explained is relatively big (eg, 6.48 %). However, when the total variance being explained is less than 5 %, all of the methods have almost no power to detect rare variants. One possible reason is that the FBAT is essentially a burden-type test. Although the proposed weighting schemes of *grv*, *lor*, and *ow* can adjust the direction of the association, it still can lose power when rare variants in a gene act in different directions on phenotypes. A variance-component based FBAT [17] could be much more powerful in such instances. Further study of what causes the power loss when detecting association on the other 4 genes with the FBAT-v–based methods is needed.

The proposed weights *grv* and *lor* are based on the effect size estimates from the marginal association. However, estimates are not stable when the variants are rare. This can be reflected from the unstable power of FBAT-v-e-grv and FBAT-v-e-lor when detecting gene *NRF1* in Table 3.
